# Pathological Findings in Chronic Inflammatory Demyelinating Polyradiculoneuropathy: A Single-Center Experience

**DOI:** 10.3390/brainsci10060383

**Published:** 2020-06-17

**Authors:** Marco Luigetti, Angela Romano, Andrea Di Paolantonio, Giulia Bisogni, Salvatore Rossi, Amelia Conte, Francesca Madia, Mario Sabatelli

**Affiliations:** 1 Fondazione Policlinico Universitario A. Gemelli IRCCS, UOC Neurologia, 00168 Rome, Italy; mluigetti@gmail.com; 2 Dipartimento di Neuroscienze, Università Cattolica del Sacro Cuore, 00168 Rome, Italy; angela.romano12@gmail.com (A.R.); andrea.dp1988@gmail.com (A.D.P.); salvatorerossi309@gmail.com (S.R.); 3 Centro Clinico NEMO-Fondazione Policlinico Universitario A. Gemelli IRCCS, 00168 Rome, Italy; giulia.bisogni@centrocliniconemo.it (G.B.); amelia.conte@centrocliniconemo.it (A.C.); 4 Fondazione Policlinico Universitario A. Gemelli IRCCS, UOC Neurofisiopatologia, 00168 Rome, Italy; francesca.madia@policlinicogemelli.it

**Keywords:** CIDP, nerve biopsy, onion bulbs, segmental demyelination, inflammatory infiltrates, regenerating clusters, axonal loss

## Abstract

Objective: Segmental demyelination is the pathological hallmark of chronic inflammatory demyelinating polyradiculoneuropathy (CIDP), but other elementary lesions are frequently observed, configuring a series of different pathological pictures. In this article, we review the pathological findings of a large series of sural nerve biopsies from our cohort of CIDP patients. Patients and Methods: Patients with CIDP who underwent nerve biopsy were retrospectively selected from those referred to the Institute of Neurology of the “Università Cattolica del Sacro Cuore” in Rome, Italy, from 1982 to February 2020. Sural nerve biopsy was performed according to standard protocols. Results: Sural nerve biopsy was performed in 43/130 CIDP patients. Demyelinating abnormalities and axonal loss were found in 67.4% and 83.7% of biopsies, respectively. Conversely, onion bulbs and inflammatory infiltrates were rare (18.6% and 4.7%, respectively). In three cases, we observed normal pathological findings. Conclusions: A pathognomonic pathological finding of CIDP cannot be established, but we confirm the utility of nerve biopsy in this setting to confirm the diagnosis (also in atypical phenotypes) and to elucidate pathogenic mechanisms.

## 1. Introduction

Chronic inflammatory demyelinating polyradiculoneuropathy (CIDP) is a clinically heterogeneous, roughly symmetric, sensory and motor neuropathy of likely immune origin [1,2,3]. Its denomination, originally coined by Dyck and co-workers [4], summarizes the main clinicopathological features of the disease, its hallmark being inflammation-mediated demyelination [1].

In its classic presentation, CIDP appears as a mainly motor neuropathy, affecting both distal and proximal muscles of the four limbs, along with sensory involvement and generalized areflexia, evolving as a monophasic, relapsing, or progressive disorder in more than two months. However, there is a remarkable heterogeneity in clinical presentation, and several variants of CIDP have thus far been described, all characterized by electrophysiological and/or histopathological features of segmental demyelination [5]. 

Demyelinating lesions are distributed in a multifocal pattern in the peripheral nervous system. The examination of sensory nerve biopsy represents a privileged instrument both for diagnostic purposes and for understanding possible pathogenic mechanisms [4,6,7,8,9,10,11,12,13,14,15,16,17].

Segmental demyelination is typically considered as the pathological hallmark of CIDP, but other elementary lesions are frequently observed, including axonal degeneration, proliferation of Schwann cells leading to the formation of “onion bulbs”, and inflammatory infiltrates. In a minority of cases, endoneural and intramyelinic edema, and axonal shrinking have been described too [4,6,7,8,9,10,11,12,13,14,15,16,17,18]. 

For many years now, macrophage-mediated demyelination has been described in CIDP. The first description by Prineas demonstrated, using electron microscopic examination of nerve samples from patients with recurrent idiopathic polyneuropathy, that myelin breakdown is initiated by macrophages penetrating Schwann cells, and in the following years, this mechanism was confirmed in other studies [7,8,9,10,11,12,13,14,15,16,17,18]. 

Recently, autoantibodies against nodes of Ranvier and paranodes have been identified, and their association with distinct subgroups of CIDP patients has been described [19].

All the described alterations can combine in many ways, configuring a series of different pathological pictures.

In this article, we review the pathological findings of a large series of sural nerve biopsies from our cohort of CIDP patients in order to underline the most frequent pathological alterations and to make a correlation with clinical findings.

## 2. Materials and Methods

### 2.1. Patients

Patients were retrospectively selected from those referred to the Institute of Neurology of the “Università Cattolica del Sacro Cuore” in Rome, Italy, from 1982 to February 2020.

Diagnosis was defined according to the European Federation of Neurological Societies and the Peripheral Nerve Society (EFNS/PNS) diagnostic criteria for CIDP [20], including mandatory clinical and electrodiagnostic criteria, potentially integrated with a set of supportive criteria. According to these criteria, patients were divided into three diagnostic categories: “definite CIDP”, “probable CIDP” or “possible CIDP” [20].

As regards the phenotype, based on clinical and electrophysiological features, patients were further divided into two classes: “typical” and “atypical” CIDP [5].

Patients with an approximately symmetrical sensory-motor neuropathy, with motor involvement grossly more prominent than the sensory one, were labeled as having a “typical CIDP”. In contrast, in the second group, we included all the patients with any of the atypical variants of CIDP so far described: distal acquired demyelinating symmetric neuropathy (DADS neuropathy); multifocal acquired demyelinating sensory and motor neuropathy (MADSAM neuropathy or Lewis–Sumner syndrome); pure motor CIDP; sensory CIDP; and focal CIDP [5].

Furthermore, according to the disease course after the initial phase, three different types of disease course were recognized: monophasic course, relapsing-remitting course, chronic progressive course [5].

### 2.2. Nerve Biopsy

Sural nerve biopsy was performed, after obtaining informed consent, as previously described [21]. Light and electron microscopy preparations, as well as teased fiber analysis, were performed according to standard methods [21].

### 2.3. Statistical Analysis 

Statistical analysis of data was performed by SPSS (Statistical Package for Social Science) version 24.0 to assess any differences between the group of patients who underwent nerve biopsy and the group of patients for which nerve biopsy was not performed (biopsy performed vs. biopsy not performed). Moreover, considering only patients who underwent nerve biopsy, statistical analysis was carried out to assess any differences based on the clinical phenotype (atypical vs. typical cases). Mann–Whitney U test and Fisher’s two-tailed exact test were used to compare numerical and nominal dichotomous variables, respectively. In case of categorical polytomous variables, a Chi-squared test was performed. Significance was set at 0.05.

### 2.4. Ethics

The study was approved by the “Fondazione Policlinico Universitario A. Gemelli IRCCS—Università Cattolica del Sacro Cuore” Ethics Committee, Rome (Prot. 23255/16 ID 1229). The study protocol conforms to the ethical guidelines of the 1975 Declaration of Helsinki (6th revision, 2008), as reflected in a priori approval by the institution‘s Human Research Committee.

## 3. Results

### 3.1. Clinical Results

**Figure 1:**
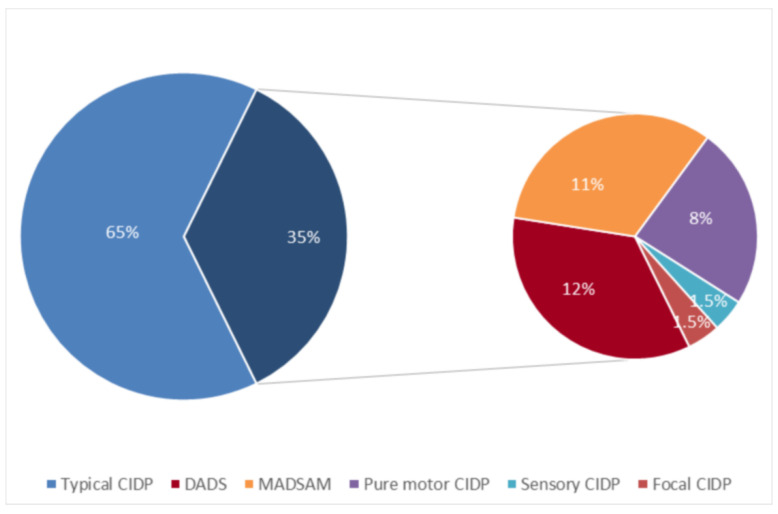
Distribution of clinical phenotypes in the entire chronic inflammatory demyelinating polyradiculoneuropathy (CIDP) cohort.

A total of 130 patients (84 males and 46 females) were diagnosed with CIDP in the period examined. Male-to-female ratio was 1.8. Mean age at onset was 48.7 years (median 54.0; standard deviation 19.3; range 7–85). Mean follow-up was 97.0 months (median 68.5; standard deviation 84.9). A typical presentation was observed in 84/130 (64.6%) patients; conversely, an atypical phenotype was observed in 46/130 (35.4%) patients (Figure 1).

Demographic and clinical characteristics of the entire CIDP cohort are summarized in Table S1.

A total of 43 out of 130 (33.1%) patients (28 males and 15 females) underwent a nerve biopsy. Male-to-female ratio was 1.9. Mean age at onset was 46.7 years (median 45.0; standard deviation 18.4; range 7–82). Mean follow-up was 139.5 months (median 130.0; standard deviation 99.1). Mean age at biopsy was 51.8 years (median 56.0; standard deviation 20.0; range 12–82). Mean time from disease onset to nerve biopsy was 68.8 months (median 25.0; standard deviation 87.4).

**Figure 2:**
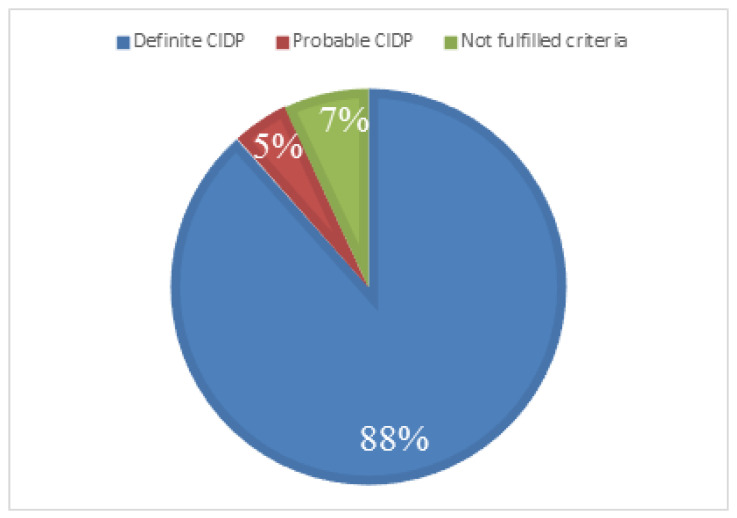
European Federation of Neurological Societies and the Peripheral Nerve Society (EFNS/PNS) diagnostic category among CIDP patients that underwent a sural nerve biopsy.

According to diagnostic criteria, 38/43 had a definite CIDP, 2/43 had a probable CIDP, 0/43 had a possible CIDP, and 3/43 did not fulfil EFNS/PNS diagnostic criteria (Figure 2).

**Figure 3:**
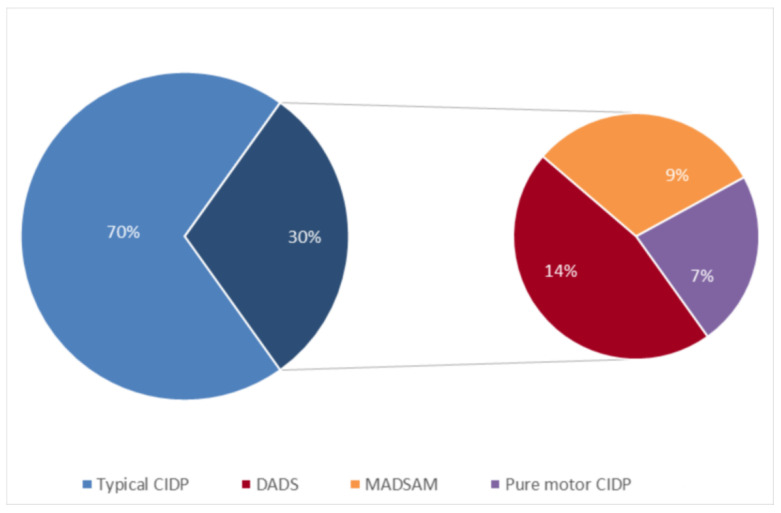
Distribution of clinical phenotypes among CIDP patients that underwent a sural nerve biopsy.

A typical presentation was observed in 30/43 (69.8%) patients (Figure 3). Mean age at biopsy was 52 years (median 54.6; standard deviation 21.2; range 12–82). Mean time from disease onset to nerve biopsy was 66.5 months (median 14.7; standard deviation 87.0).

An atypical phenotype was observed in 13/43 (30.2%) patients (Figure 3). Mean age at biopsy was 51.5 years (median 56.5; standard deviation 17.9; range 13–82). Mean time from disease onset to nerve biopsy was 74.3 months (median 34.7; standard deviation 91.7).

Demographic and clinical characteristics of CIDP patients who underwent nerve biopsy and their comparison with the remaining cohort are summarized Table S2.

### 3.2. Pathological Results

**Figure 4:**
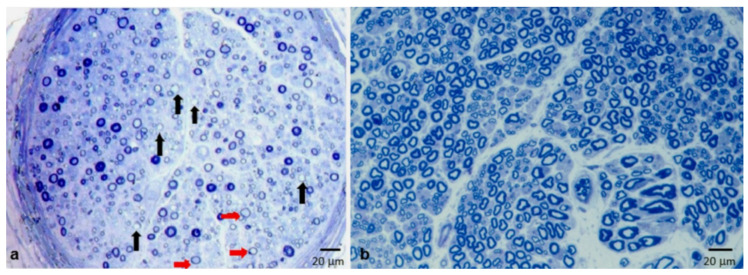
Semithin section stained with toluidine blue. Sural nerve biopsy performed in a 66-year-old man with “typical” CIDP (**a**), and sural nerve biopsy from a normal subject matched per age (**b**). A moderate reduction of myelinated fibers is shown (a). Many fibers (75%) have a thin myelin sheath if compared with axon diameter (red arrows in (a)); few fibers (4%) are devoid of myelin sheath (“naked axons”, black arrows in (a)).

**Figure 5:**
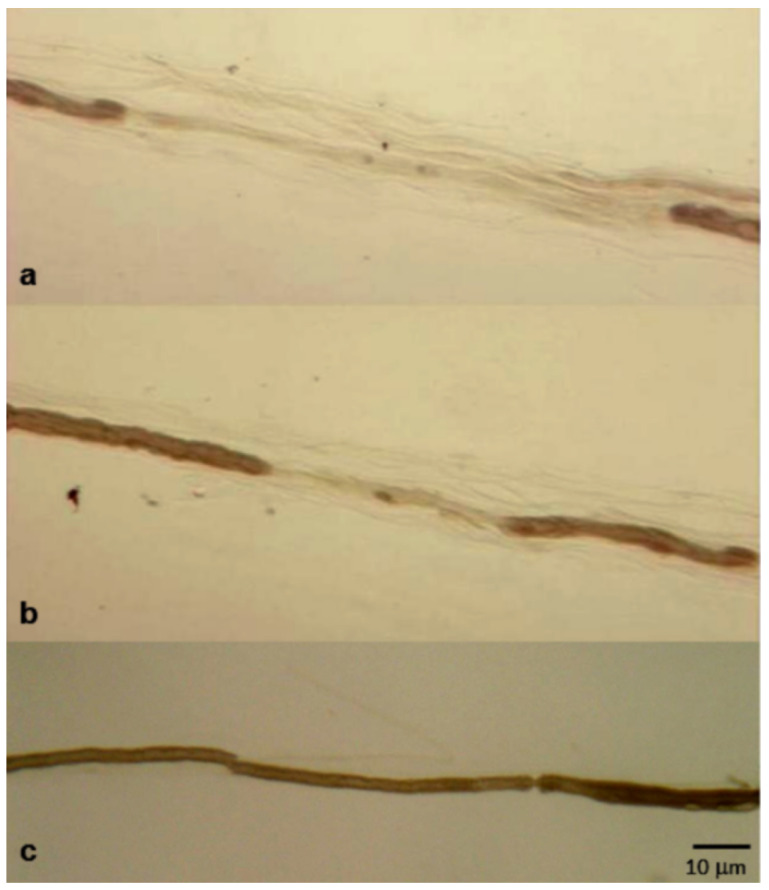
Teased fibers analysis from sural nerve biopsy performed in a 65-year-old man with “atypical” CIDP (multifocal acquired demyelinating sensory and motor neuropathy (MADSAM)) (**a**,**b**) and from sural nerve biopsy performed in an age-matched control with an axonal polyneuropathy (**c**). Segmental demyelination, found in about 40% of fibers, is shown (a,b).

Obvious demyelinating abnormalities in both semithin sections and teased fiber analysis were found in 16 biopsies (37.2%). Conversely, demyelination was detected only in semithin sections (fibers with very thin or absent myelin sheath) in seven cases (16.3%) (Figure 4a), and only in teased fiber analysis in a further six patients (13.9%) (Figure 5a,b). Remyelination was observed on teased fiber analysis in nine cases (20.9%).

**Figure 6:**
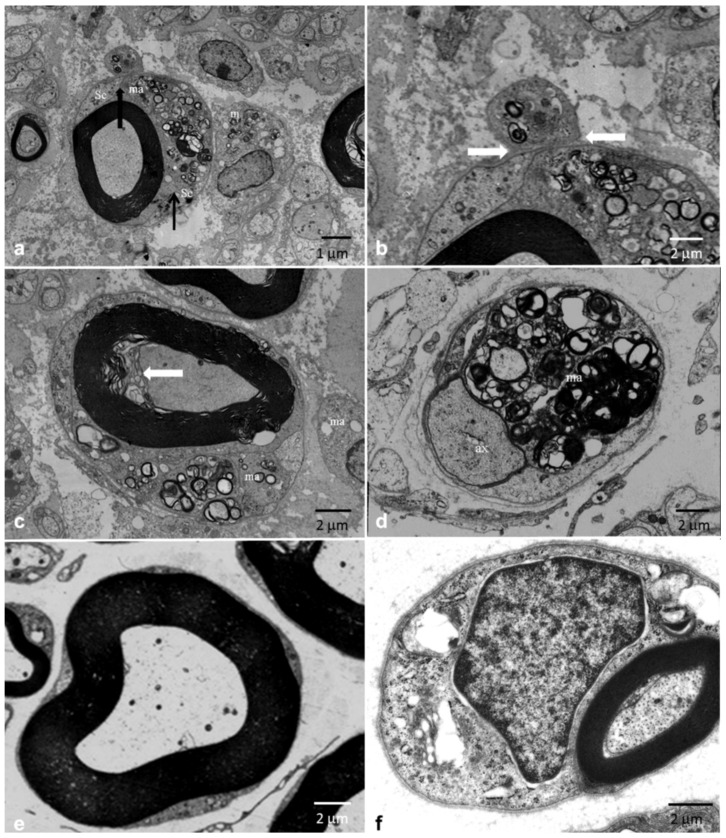
Sural nerve biopsy from a 37-year-old man with “typical” CIDP (**a**–**d**) and normal age-matched controls (**e**,**f**). Electron microscopy. Macrophage-mediated demyelination. (**a**) A fiber with normal myelin appearance wrapped in a macrophage (ma) is observed; in the macrophage’s cytoplasm, there are numerous myelinated figures, expression of an early phase of the demyelination process. The arrows indicate the demarcation between the cytoplasm of the Schwann cell (Sc) and that of the macrophage. Separated from the fiber, another macrophage (m) participates in the “digestion” of the myelin. (**b**) Greater enlargement of a detail of (a). The macrophage has broken off the basement membrane (arrows) to attack the myelin. (**c**) Myelin begins to decompact and has a “beehive” (arrow) appearance. Also, in this case, the macrophage (ma) actively participates in the process of destruction of the myelin sheath. (**d**) Axon (ax) appears completely devoid of the myelin sheath. Also, in this case, there is a macrophage (ma) loaded with myelin debris. (**e**) Normal myelinated fiber. (**f**) Normal myelinated fiber surrounded by Schwann cell.

Electron microscopy confirmed the alterations seen on light microscopy and in some cases showed macrophages loaded with myelin debris invading the basal lamina of myelinated fibers (Figure 6a–d). 

In three patients, evidence of demyelination and/or remyelination was not evaluable because of the severe axonal loss. In the remaining 11 patients (25.6%), there was not any feature suggestive of either demyelination or remyelination.

**Figure 7:**
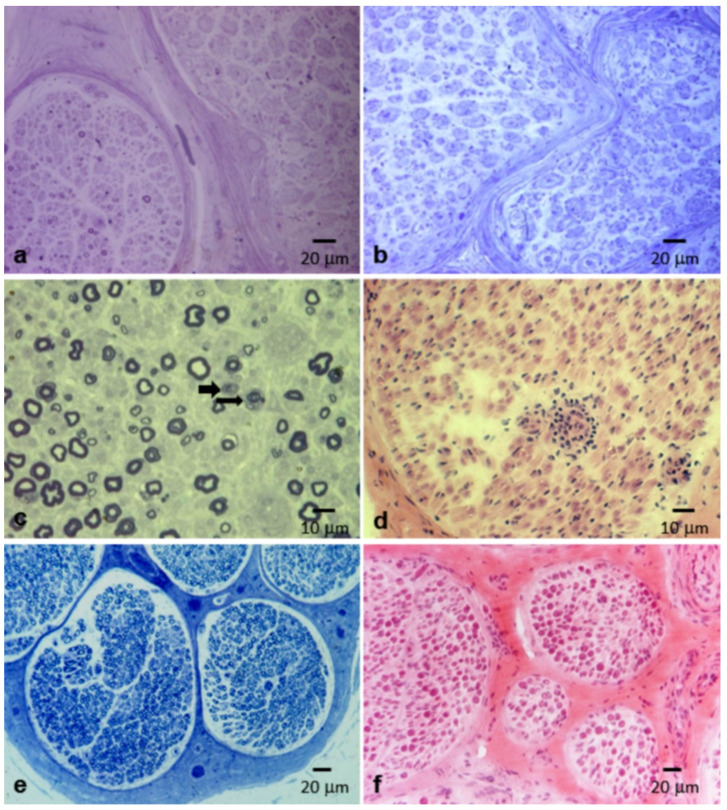
Different pathological alterations in CIDP (**a**–**c**) and normal age-matched control (**e**,**f**). (**a**–**c**) Semithin section from CIDP patients stained with toluidine blue. (**a**) Sural nerve biopsy from a 64-year-old woman with “typical” CIDP. Two contiguous fascicles with completely different aspects: in one, only a loss of myelin fibers is observed; while in the other, fibers are extremely reduced in number and are all surrounded by onion bulbs. (**b**) Sural nerve biopsy from a 47-year-old woman with “typical” CIDP: marked reduction of myelinated fibers, all surrounded by onion bulbs, is observed. (**c**) Sural nerve biopsy from a 65-year-old man with “atypical” CIDP (MADSAM, shown in Figure 5a,b): slight reduction of myelin fibers and active axonal degenerations (arrows) are observed. (**d**) Hematoxylin and eosin (H&E) staining from a CIDP patient. Sural nerve biopsy from a 53-year-old man with “typical” CIDP: an endoneural inflammatory infiltrate is present. (**e**) Semithin section from a normal subject stained with toluidine blue. (**f**) H&E staining from a normal subject.

Onion bulbs and inflammatory infiltrates, considered as other typical features of the disease, were present only in eight (18.6%) and two (4.7%) patients, respectively (Figure 7a–d).

**Figure 8:**
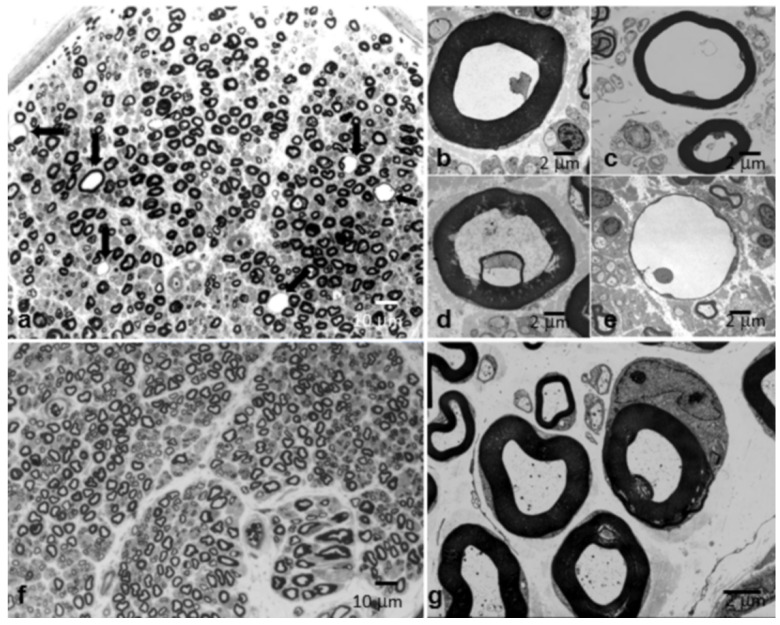
Sural nerve biopsy from a 58-year-old man with “typical” CIDP (**a**). Semithin sections stained with toluidine blue (**a**). Numerous fibers appear vacuolated (arrows in (a)) and show a distension and thinning of the myelin sheath (a). Electron microscope (**b–e**). Sural nerve biopsies from a 63-year-old woman with “typical” CIDP (**b**); from a 58-year-old man with “typical” CIDP (shown in (a)) (**c**); from a 61-year-old man with “typical” CIDP (**d**); and from the 65-year-old man with “atypical” CIDP (MADSAM) shown in Figure 5a,b and Figure 7c (**e**). Intramyelinic edema appears as a finely granular fluid material that relaxes and deforms the myelin sheath. The fibers affected have a swollen appearance with a markedly increased diameter: myelin decompaction occurs at the level of the major dense line, and the axons invariably show a marked reduction in diameter, as shrunk by osmotic mechanisms. (**f**) Semithin section stained with toluidine blue from a normal age-matched control. (**g**) Electron microscope. Myelinated fibers from a normal age-matched control.

Subperineural edema was detected in four patients (9.3%), and endoneural edema in another patient (2.3%). Marked myelin swelling due to intramyelinic edema was observed in three other cases (7.0%) (Figure 8a–e).

In the great majority of nerve specimens (36 out of 43 nerve biopsies, 83.7%), we detected a variable degree of axonal loss (mild in 10 cases, moderate in 11, severe in 15), sometimes with focal distribution (Figure 4a). Wallerian degenerations were noted in 22 cases (51.2%), and regenerating clusters were observed in 26 biopsies (60.5%) (Figure 7c).

Detailed pathological findings are summarized in Table 1. 

In our cohort, a similar percentage of biopsies was performed in patients with typical and atypical phenotypes, and no specific pathological alteration was predominant in one group than in the other. Comparisons between pathological findings among the patients who underwent nerve biopsy based on the clinical phenotype are reported in Table S3.

## 4. Discussion

Our series confirms that segmental demyelination is the pathological hallmark of CIDP, together with other elementary lesions, as widely reported in the literature on nerve biopsies and in rare autopsy studies (Table 2) [22]. Importantly, these alterations may combine in many different ways, configuring a series of different pathological pictures in CIDP (Table 1) [4,6,7,8,9,10,11,12,13,14,15,16,17].

Several factors contribute to such variability. Firstly, the observed picture on nerve biopsy represents only a snapshot of the cascade of events occurring over time. Therefore, the type of lesions observed is strictly dependent on the disease phase in which the sampling is performed. Secondly, CIDP is a multifocal disorder where the spatial distribution of the lesions follows the laws of variability and unpredictability typical of stochastic phenomena, including inflammatory processes [23]. The nerves commonly accessible to the biopsy are the sural and the superficial peroneal nerves, and their variable involvement depends on the casual spatial distribution of the lesions throughout the peripheral nervous system. Finally, it should be considered that pathological heterogeneity could reflect different mechanisms of immune responses involved in CIDP. Supporting this hypothesis is the recent discovery that some immune-mediated neuropathies, usually classified as CIDP, are caused by antibodies that alter the ultrastructural organization of the paranodes without inflammation or overt demyelination [24,25].

Segmental demyelination is characterized by the destruction of the myelin sheath which generally involves short (less than one internode long) segments, while the axonal structures remain intact. In our series, demyelination was found in 67% of patients (Table 1) [4,6,7,8,9,10,11,12,13,14,15,16,17].

Demyelinating phenomena can be observed in light microscopy and/or electron microscopy, but teased fiber examination allows a more accurate identification and quantification of these phenomena (Figure 5a,b) [4]. 

The American Academy of Neurology (AAN) commission suggested quantitative criteria to establish “unequivocal evidence of demyelination and/or remyelination” in nerve biopsies of patients with CIDP: at least 12% of 50 dissociated fibers, minimum of four internodes each, must have these alterations, or by electron microscopy, a minimum of five fibers must show demyelination [3].

Although demyelination represents the prevalent and probably primary event of CIDP, pathological examination shows that, in most patients, this lesion is associated with a significant loss of fibers (Figure 4a). In our series, we reported a variable degree of axonal loss in almost 85% of biopsies (Table 1). 

Axonal involvement can represent an “innocent by-stander” effect, a non-specific consequence of an inflammatory process that primarily affects myelin. However, it should be borne in mind that the distinction between axonal and demyelinating pathologies, while retaining an undoubted value in the diagnosis and classification of neuropathies, is an artificial simplification since neuritis and Schwann cells form a morpho-functional unit in which the two components are strictly interdependent [26].

A “pure” demyelinating disease, in which the nerve biopsy showed only myelin destruction without the involvement of the axons, was observed in only 5% in our cohort (two patients) [4,6,7,8,9,10,11,12,13,14,15,16,17]. 

It is important to underline that, in about 25% of our cohort, the nerve biopsy does not show clear demyelinating phenomena but only a loss of fibers, associated or not with axonal degeneration in the active phase. This finding may be a consequence of the multifocality of the disease. Fiber loss will be the only alteration when active inflammation, responsible for demyelination and destruction of the axons, occurs in the segments proximal to the site of the biopsy sample. Confirming this data, in all patients with pathological evidence of “axonal” neuropathy from our cohort, neurophysiological investigations (which, unlike biopsy, have the undoubted advantage of allowing an extensive study of the nerves) show signs of demyelination. 

However, rare cases have been reported in which both the pathological examination of the nerve and nerve conduction studies showed an exclusively axonal involvement [26]. One possible explanation is that there is a form of chronic inflammatory polyradiculoneuropathy in which the immune process has other targets rather than myelin, similarly to what was observed for the axonal forms of Guillain-Barré syndrome [27]. 

The hypothesis of an inflammatory nature for CIDP is related to the observation that inflammatory cells, namely macrophages and T cells, actively participate in the pathogenesis of the disease [28]. However, clear inflammatory infiltrates on light microscopy are found only in a minority of cases (Table 1). We confirmed this data with inflammatory infiltrates found in only 5% of biopsies in our cohort (Figure 7d). The role of macrophages in demyelination can be better observed using electron microscopy, which shows that the “stripping” of myelin by macrophages likely represents the primary event [29]. Accordingly, our findings by electron microscopy showed that macrophage-mediated demyelination is a central mechanism in CIDP. In Figure 6a,b, a macrophage that penetrated below the basement membrane of a myelinated fiber can be seen. The abundant myelin figures in its cytoplasm, in the presence of a normal myelin sheath, indicate that the macrophage was initiating the destruction of the myelin and was not simply scavenging myelin debris. In a subsequent phase, the myelin sheath undergoes degenerative phenomenon, with an initial decompaction (Figure 6c) until a complete disruption (Figure 6d). 

In one patient in our cohort, we detected antibodies against Contactin-1 [30]: the pathological picture of this patient revealed a mild axonal loss with occasional Wallerian degeneration without demyelination, consistent with the reported data [31].

In a variable percentage (Table 1), the morphological picture is dominated by phenomena of Schwann cell proliferation, leading to the formation of the classic “onion bulbs” (Figure 7a,b), and to an increase of the fascicular area, which corresponds to a macroscopic enlargement of the nerves, appreciable on palpation and, in some cases, with neuroimaging [32,33]. In our experience, onion bulbs are observed only in a minority of cases (about 20%). Onion bulbs are considered an unspecific reaction of the Schwann cell and fibroblasts to repeated phenomena of demyelination and remyelination, but it is not clear why this finding is absent in the majority of cases, including those with a long history of disease (Figure 7a).

In our cohort, 7% of biopsies were completely normal, a percentage similar to that reported in the literature, ranging from 10% to 20% of cases. This can be explained once again by the multifocal nature of CIDP, in which the nerve sample may not show any alterations because it is spared from the inflammatory process by chance. Furthermore, normal aspects of nerve biopsy, when associated with the absence of clinical and electrophysiological sensory involvement, occur in pure motor CIDP, which has been suggested to represent a distinct nosological entity [5,34,35]. Finally, the absence of lesions in nerve biopsy can be explained with the hypothesis that in some forms of CIDP the loss of function of nerve fibers is not attributable to morphological changes, as demyelination, axonal degeneration or paranodal dismantling, but to the failure of conduction caused by antibodies blocking ion channels [24,29]. 

In some patients [36], the nerve biopsy shows a vacuolization of fibers due to an accumulation of water and proteinaceous material inside the myelin sheath suggestive of intramyelinic edema (IE). Under optical microscopy, the fibers affected by the IE have a swollen appearance with a markedly increased diameter (Figure 8). Under electron microscopy (Figure 8), myelin decompaction occurs at the level of the major dense line, and the axons invariably show a marked reduction in diameter, as shrunk by osmotic mechanisms. Our data, and studies in experimental neuropathies [18,37], indicate that IE may represent a transient phase of a demyelinating process and the possibility that IE may per se impair impulse propagation seems likely. The fact that IE has been rarely reported in human pathology suggests that this elementary lesion may be specific for some CIDP subtypes or may represent a short-lived aspect of demyelinating neuropathies [36].

## 5. Conclusions

In conclusion, we confirm that nerve biopsy may be helpful in the diagnosis of CIDP and may probably contribute to improve our understanding of pathogenetic mechanisms and to identify proper treatments.

## Figures and Tables

**Table 1:** Detailed pathological finding of 43 nerve biopsies.

	Count (%)
**Final histopathological findings**	
Demyelinating	2 (4.7%)
Mixed	29 (67.4%)
Axonal	9 (20.9%)
Normal	3 (7.0%)
**Evidence of demyelination**	
Absent	11 (25.6%)
Only by teased fiber analysis	6 (13.9%)
Only by semithin sections analysis	7 (16.3%)
Both by semithin sections and teased fiber analysis	16 (37.2%)
Not evaluable	3 (7.0%)
**Evidence of remyelination by teased fiber analysis**	9 (20.9%)
**Onion bulbs**	8 (18.6%)
**Loss of fibers**	
Absent	7 (16.3%)
Mild	10 (23.2%)
Moderate	11 (25.6%)
Severe	15 (34.9%)
**Axonal degeneration**	22 (51.2%)
**Regeneration clusters**	26 (60.5%)
**Inflammatory infiltrates**	2 (4.7%)
**Vasa nervorum abnormalities**	0 (0%)
**Intramyelinic edema**	3 (7.0%)
**Endoneural or subepineural edema**	5 (11.6%)

**Table 2:** Pathological alterations (%) in different reported cohorts of CIDP.

Reference	No. of Biopsies	Demyelination	Axonal Loss/Degeneration	Onion Bulbs	Inflammatory Infiltrates	Normal Pathology
Dyck et al., 1975 [4]	26	23.4	100	15	19	0
Prineas et al., 1976 [6]	26 *	80	30	39	NR	20
Barohn et al., 1989 [7]	56	60.7	33.9	NR	10.7	17.9
Krendel et al., 1989 [8]	14	50	NR	36	29	NR
Azulay et al., 1992 [9]	20	90	55	35	15	10
Matsumuro et al., 1994 [10]	9	100	88.9	22	22.2	0
Gorson et al., 1997 [11]	18	39	61	5	17	17
Rizzuto et al., 1998 [12]	105	100	85	48	25	0
Bouchard et al., 1999 [13]	95 ^#^	72	47	18	4	2
Vallat et al., 2003 [14]	8 **	100	100	NR	75	0
Kulkarmi et al., 2010 [15]	46	82.8	56.6	28.3	58.7	0
Piccione et al., 2016 [16]	26 ^#^	46.0	69.2	38.5	61.5	0
Ikeda et al., 2019 [17]	106	22.8	8.1	NR	29.7	NR
Luigetti et al. (this paper)	43	67.4	83.8	18.6	4.7	7.0

* 25 sural nerve and 1 radial nerve; ** 7 sural nerve and 1 radial nerve; ^#^ nerve biopsied not clearly specified. In the remaining papers all biopsies are sural nerve. NR, not reported.

## Supplementary Materials

The following are available online at https://www.mdpi.com/2076-3425/10/6/383/s1, Table S1: Demographic and clinical characteristics of the cohort of study, Table S2: Demographic and clinical characteristics of CIDP patients who underwent nerve biopsy and statistical analysis to assess any differences between this group of patients and the group of patients for which nerve biopsy was not performed, Table S3: Statistical analysis to assess any differences among the patients who underwent nerve biopsy based on the clinical phenotype.
