# Supplementary material for: Pathological Findings in Chronic Inflammatory Demyelinating Polyradiculoneuropathy: A Single-Center Experience
Source: Brain Sci. 2020 Jun 17;10(6):383. doi: 10.3390/brainsci10060383 (PMC7349397; doi:10.3390/brainsci10060383)
Supplement: Supplementary file 1 [file brainsci-10-00383-s001.zip › brainsci-814673-SI/Table S1.pdf]

Supplementary Table S1. Demographic and clinical characteristics of the whole cohort of study.

	**Count (%)**	**Mean (SD)**	**n**
**Males/Females**	84 (64.6%)/46 (35.4%)		130
**Age at disease onset (years)**		48.69 (19.25)	130
**Disease duration (months)**		130.96 (150.65)	130
**Follow-up (months)**		96.98 (84.93)	130
**Disease onset**			130
**Chronic**	89 (68.5%)		
**Acute**	24 (18.5%)		
**Subacute**	17 (13.1%)		
**Phenotype**			130
**Typical CIDP**	84 (64.6%)		
**DADS**	16 (12.3%)		
**MADSAM**	15 (11.5%)		
**Pure motor CIDP**	11 (8.5%)		
**Sensory CIDP**	2 (1.5%)		
**Focal CIDP**	2 (1.5%)		
**Disease course**			130
**Monophasic**	26 (20.0%)		
**Relapsing**	30 (23.1%)		
**Chronic progressive (or chronic active)**	74 (56.9%)		
**EFNS/PNS electrodiagnostic criteria**			130
**Definite**	78 (60.0%)		
**Probable**	18 (13.8%)		
**Possible**	19 (14.6%)		
**Not fulfilled**	15 (11.5%)		
**EFNS/PNS diagnostic category**			130
**Definite CIDP**	103 (79.2%)		
**Probable CIDP**	11 (8.5%)		
**Possible CIDP**	0 (0.0%)		
**Not fulfilled**	16 (12.3%)		

Legend to the table: SD, standard deviation; CIDP, Chronic Inflammatory Demyelinating Polyneuropathy; DADS, Distal Acquired Demyelinating Symmetric Neuropathy; MADSAM, Multifocal Acquired Demyelinating Sensory And Motor Neuropathy; EFNS/PNS, European Federation of Neurological Societies/Peripheral Nerve Society.
