# Supplementary material for: Pathological Findings in Chronic Inflammatory Demyelinating Polyradiculoneuropathy: A Single-Center Experience
Source: Brain Sci. 2020 Jun 17;10(6):383. doi: 10.3390/brainsci10060383 (PMC7349397; doi:10.3390/brainsci10060383)
Supplement: Supplementary file 1 [file brainsci-10-00383-s001.zip › brainsci-814673-SI/Table S2.pdf]

Supplementary Table S2. Demographic and clinical characteristics of CIDP patients who underwent nerve biopsy and statistical analysis to assess any differences between this group of patients and the group of patients for which nerve biopsy was not performed.

	Nerve biopsy not performed (n=87)			Nerve biopsy performed (n=43)			FET	CS	MWUT
	**count (%)**	**mean (SD)**	**n**	**count (%)**	**mean (SD)**	**n**			
**Males/Females**	56 (64.4%)/31 (35.6%)		87	28 (65.1%)/15 (34.9%)		43	NS		
**Age at disease onset (years)**		49.70 (19.70)	87		46.65 (18.35)	43			NS
**Disease duration (months)**		108.20 (158.14)	87		177.02 (123.53)	43			P=0.001
**Follow-up (months)**		75,99 (68.36)	87		139,47 (99.12)	43			P=0.001
**Disease onset**			87			43		NS	
**Chronic**	60 (69.0%)			29 (67.4%)					
**Acute**	16 (18.4%)			8 (18.6%)					
**Subacute**	11 (12.6%)			6 (14.0%)					
**Phenotype**			87			43		NS	
**Typical CIDP**	54 (62.1%)			30 (69.8%)					
**DADS**	10 (11.5%)			6 (14.0%)					
**MADSAM**	11 (12.6%)			4 (9.3%)					
**Pure motor CIDP**	8 (9.2%)			3 (7.0%)					
**Sensory CIDP**	2 (2.3%)			0 (0%)					
**Focal CIDP**	2 (2.3%)			0 (0%)					
**Disease course**			87			43		NS	
**Monophasic**	21 (24.1%)			5 (11.6%)					
**Relapsing**	20 (23.0%)			10 (23.3%)					
**Chronic progressive**	46 (52.9%)			28 (65.1%)					
**Electrophysiological features**			87			43		NS	
**Demyelinating**	12 (13.8%)			7 (16.3%)					
**Mixed**	73 (83.9%)			36 (83.7%)					
**Axonal**	2 (2.3%)			0 (0%)					
**EFNS/PNS electrophysiological criteria**			87			43		NS	
**Definite**	49 (56.3%)			29 (67.4%)					
**Probable**	13 (14.9%)			5 (11.6%)					
**Possible**	13 (14.9%)			6 (14.0%)					
**Not fulfilled**	12 (13.8%)			3 (7.0%)					
**EFNS/PNS diagnostic category**			87			43		NS	
**Definite CIDP**	65 (74.7%)			38 (88.4%)					
**Probable CIDP**	9 (10.3%)			2 (4.7%)					
**Possible CIDP**	0 (0%)			0 (0%)					
**Not fulfilled**	13 (14.9%)			3 (7.0%)					

Legend to the table: SD, standard deviation. FET, Fishers' exact test; Chi-squared test; MWUT, Mann-Whitney U Test; CIDP, Chronic Inflammatory Demyelinating Polyneuropathy; DADS, Distal Acquired Demyelinating Symmetric Neuropathy; MADSAM, Multifocal Acquired Demyelinating Sensory And Motor Neuropathy; EFNS/PNS, European Federation of Neurological Societies/Peripheral Nerve Society.
