# Supplementary material for: Pathological Findings in Chronic Inflammatory Demyelinating Polyradiculoneuropathy: A Single-Center Experience
Source: Brain Sci. 2020 Jun 17;10(6):383. doi: 10.3390/brainsci10060383 (PMC7349397; doi:10.3390/brainsci10060383)
Supplement: Supplementary file 1 [file brainsci-10-00383-s001.zip › brainsci-814673-SI/Table S3.pdf]

**Supplementary Table S3.** Statistical analysis to assess any differences among the patients who underwent nerve biopsy based on the clinical phenotype.

| | Atypical (n=13) | | | Typical (n=30) | | | FET | CS | MWUT |
|---|---|---|---|---|---|---|---|---|---|
| | **count (%)** | **mean (SD)** | **n** | **count (%)** | **mean (SD)** | **n** | | | |
| **Age at the time of nerve biopsy (years)** | | 51.46 (17.92) | 13 | | 51.97 (21.20) | 30 | | | NS |
| **Time from disease onset to nerve biopsy (months)** | | 74.31 (91.69) | 13 | | 66.47 (87.02) | 30 | | | NS |
| | | | | | | | | | |
| **Final histopathological findings** | | | 13 | | | 30 | | NS | |
| **Demyelinating** | 0 (0%) | | | 2 (6.7%) | | | | | |
| **Mixed** | 10 (76.9%) | | | 19 (63.3%) | | | | | |
| **Axonal** | 2 (15.4%) | | | 7 (23.3%) | | | | | |
| **Normal** | 1 (7.7%) | | | 2 (6.7%) | | | | | |
| | | | | | | | | | |
| **Evidence of demyelination by semithin sections and/or teased fibre analysis** | | | 13 | | | 30 | | NS | |
| **Absent** | 2 (15.4%) | | | 9 (30.0%) | | | | | |
| **Only by teased fibre analysis** | 2 (15.4%) | | | 4 (13.3%) | | | | | |
| **Only by semithin sections analysis** | 4 (30.8%) | | | 3 (10.0%) | | | | | |
| **Both by semithin sections and teased fibre analysis** | 5 (38.5%) | | | 11 (36.6%) | | | | | |
| **Not evaluable** | 0 (0%) | | | 3 (10.0%) | | | | | |
| | | | | | | | | | |
| **Macrophage-mediated demyelination (TEM)** | 1 (7.7%) | | 13 | 0 (0%) | | 30 | NS | | |
| **Evidence of remyelination by teased fibre analysis** | 3 (23.1%) | | 13 | 6 (20.0%) | | 30 | NS | | |
| **Onion bulbs** | 1 (7.7%) | | 13 | 7 (23.3%) | | 30 | NS | | |
| | | | | | | | | | |
| **Loss of fibres** | | | 13 | | | 30 | | NS | |
| **Absent** | 1 (7.7%) | | | 6 (20.0%) | | | | | |
| **Mild** | 3 (23.1%) | | | 7 (23.3%) | | | | | |
| **Moderate** | 7 (53.8%) | | | 4 (13.3%) | | | | | |
| **Severe** | 2 (15.4%) | | | 13 (43.3%) | | | | | |
| | | | | | | | | | |
| **Axonal degeneration** | 8 (61.5%) | | 13 | 14 (46.7%) | | 30 | NS | | |
| **Regeneration clusters** | 8 (61.5%) | | 13 | 18 (60.0%) | | 30 | NS | | |
| **Inflammatory infiltrates** | 1 (7.7%) | | 13 | 1 (3.3%) | | 30 | NS | | |
| **Vasa nervorum abnormalities** | 0 (0%) | | 13 | 0 (0%) | | 30 | NP | | |
| **Intramielinic edema** | 1 (7.7%) | | 13 | 2 (6.7%) | | 30 | NS | | |
| **Endoneural or subepineural edema** | 2 (15.4%) | | 13 | 3 (10%) | | 30 | NS | | |

***Legend to the table:*** Abbreviations: SD, standard deviation. FET, Fishers' exact test; CS, Chi-squared test; MWUT, Mann-Whitney U Test; TEM, Transmission Electron Microscope.
